# Hepatic Transplantation Raises Concern for Vascular Infrastructure Compromise: A Case Study of Debilitating Arteriovenous Malformation

**DOI:** 10.7759/cureus.18518

**Published:** 2021-10-05

**Authors:** Elizabeth D Liu, Antonia Nituleasa, Ryan F Amidon, Christ Ordookhanian, Paul Kaloostian

**Affiliations:** 1 Biochemistry, University of California Riverside, Riverside, USA; 2 Medical Physiology, Medical College of Wisconsin, Milwaukee, USA; 3 Medicine, Medical College of Wisconsin, Milwaukee, USA; 4 Medicine, University of California, Riverside, USA; 5 Neurological Surgery, Riverside Community Hospital, Riverside, USA; 6 Neurological Surgery, Paul Kaloostian M.D. Inc., Riverside, USA

**Keywords:** subarachnoid hemorrhage, intraventricular hemorrhage, intracerebral hemorrhage, steroids, brainstem reflexes, intracranial hemorrhage, ruptured arteriovenous malformation, liver transplant

## Abstract

The post-operative realm for hepatic transplant patients presents many challenges, but of them all, we take a deeper dive into an increased risk of associated cerebrovascular events. Cerebrovascular diseases, such as cerebral arteriovenous malformation (AVM), are a leading cause of death following a liver transplant. We present a unique case of a liver transplant patient who presented with no brainstem reflexes three months into the post-transplant period. Imaging studies revealed a ruptured AVM within the foramen magnum and cervicomedullary junction, as well as substantial cerebral hemorrhage. While establishing the exact cause of the AVM is not as trivial as it may appear, side effects associated with post-transplantation management regimens and possible congenital factors do shed some light on notable considerations. Given the potential damage associated with ruptured AVMs, poor patient outcomes are unfortunately not as rare as one would hope. This case highlights a rare but highly possible occurrence for cerebrovascular complications, specifically AVM rupture linked to liver transplantation and the systemic changes associated with a procedure as invasive as liver transplantation.

## Introduction

Liver transplantation (LT) is a life-saving procedure that removes a patient’s diseased liver and replaces it with a whole or partial healthy liver from a donor. A retrospective study examining the outcomes of 1,956 LT patients noted a significantly increased prevalence of cerebral arteriovenous malformations (AVMs) in LT patients (0.26%) in comparison to a healthy adult control group (0.06%) [1]. As the second most common organ transplant type, with over 8,000 procedures performed annually, this increased risk of AVM following LT highlights the importance of identifying potential factors or treatment components likely associated with the onset of cerebrovascular events in post-LT patients [2].

Following an LT, efforts to maintain graft survival are achieved through immunosuppression treatment protocols. Lifelong immunosuppression is required to dampen the recipient’s immune system response to the donated organ and is critical in preventing organ rejection [3]. The five most commonly used post-LT immunosuppressive drug classes: corticosteroids, calcineurin inhibitors (CNIs), inhibitors of mammalian target of rapamycin (mTOR), antimetabolites, and monoclonal antibody therapy [3,4]. Each class targets different aspects of the immune response and modern post-LT immunosuppressant regimens typically contain a calcineurin inhibitor in combination with an antimetabolite or an mTOR inhibitor class drug [3,4].

While improvements to immunosuppressant options have improved long-term survival rates of LT recipients, the leading causes of mortality in long-term LT recipient survivors are associated with sustained usage of immunosuppressants [4]. The usage of immunosuppressive agents as part of the post-operative treatment regimens is associated with a myriad of adverse side effects [4]. In combination with an underlying predisposition to hypertension, diabetes, weight gain, and dyslipidemia found in the LT patient population, immunosuppressants may serve to further increase the risk of cerebrovascular events [1,4]. Continual immunosuppression and resulting co-morbidities are suggested to be primary contributors to the increased risk of cardiovascular and cerebrovascular diseases [1].

In this case, we present the case of a cerebrovascular event in an LT recipient with serious neurological complications and a poor outcome. A patient presented with no brainstem reflexes three months after an uncomplicated LT procedure. Later imaging studies revealed extensive cerebrovascular complications and hemorrhaging. These findings, combined with poor surgical candidacy, resulted in the pronunciation of brain death. 

## Case presentation

**Figure 1:**
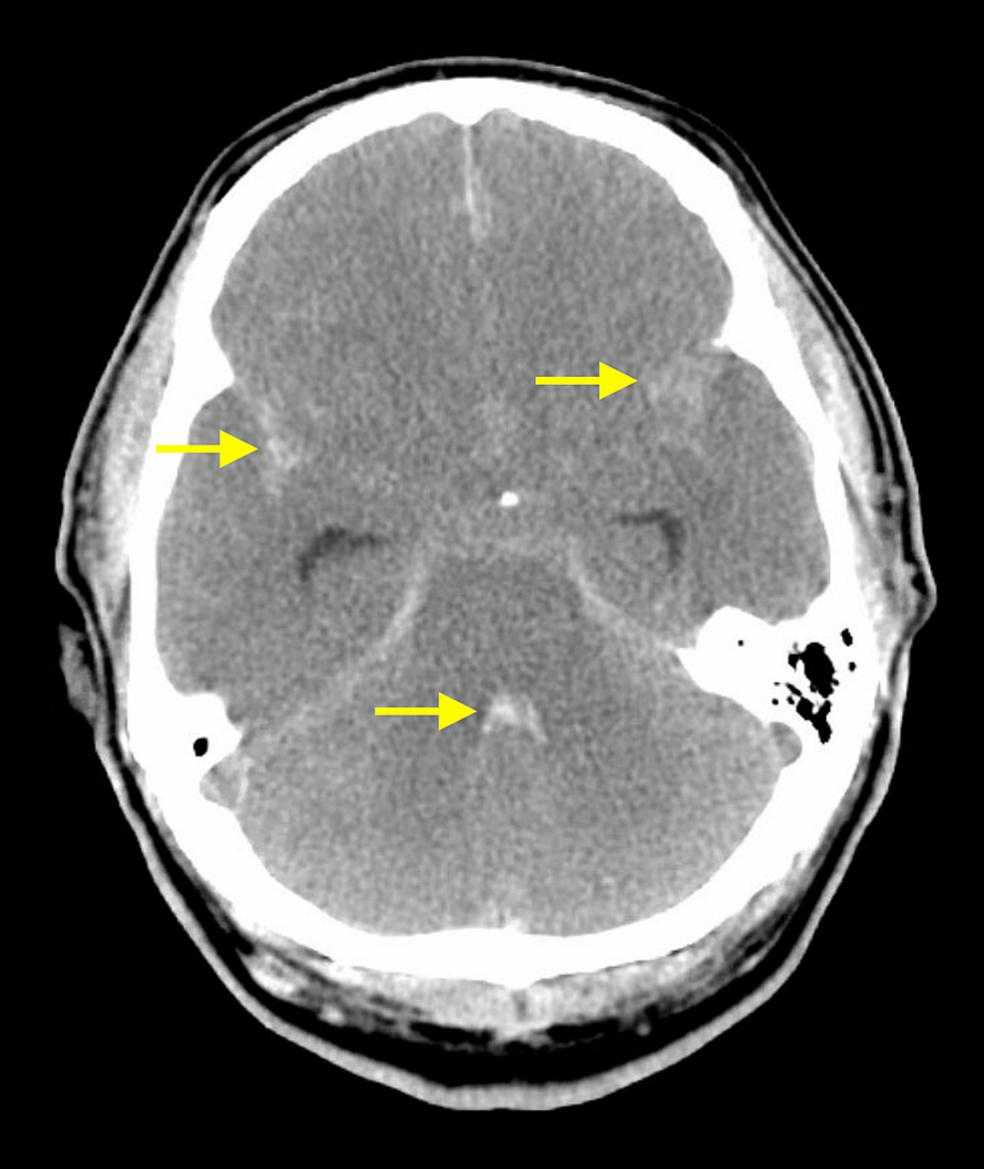
CT imaging reveals hemorrhage within the fourth ventricle (center arrow) and within the anterior subarachnoid spaces bilaterally (lateral arrows) in close proximity to the Sylvian fissure. CT: computed tomography.

A 55-year-old Hispanic male was found at home without brainstem reflexes by a family member who reported symptomology of paralysis, which caused alarm and summoning of emergency medical services. Our patient's medical history is notable for an uncomplicated liver transplant three months earlier and was placed on steroids without anticoagulation medication. A neurosurgical evaluation found no brainstem reflexes in both upper and lower extremities, including no movement in response to painful stimuli or vibratory sensations. A computed tomography (CT) scan of the head showed large intracerebral hemorrhage (ICH), an intraventricular hemorrhage in the third and fourth ventricles, and a subarachnoid hemorrhage (SH) (Figure 1).

**Figure 2:**
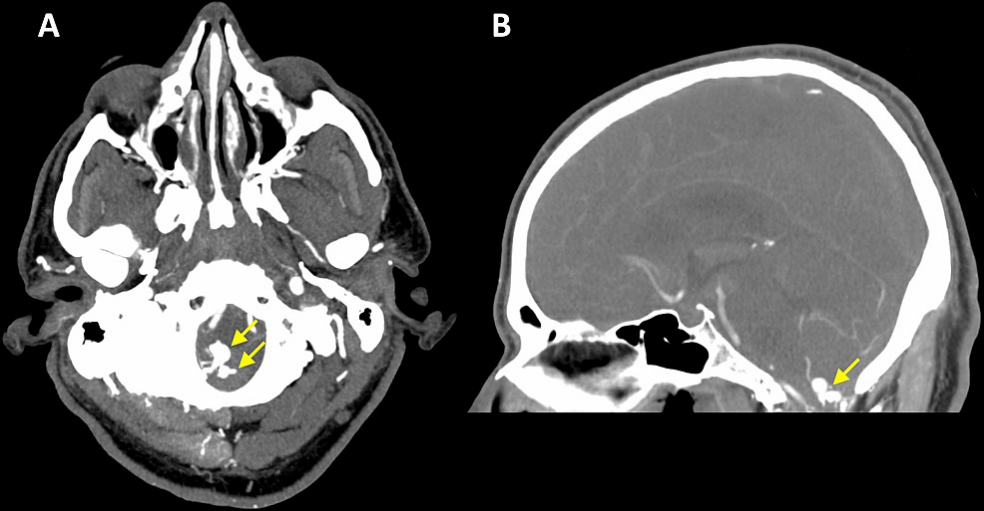
(A) Transverse CT angiogram demonstrating AVM nidus (arrow) within the foramen magnum. (B) Sagittal CT angiogram demonstrating AVM nidus (arrow) within the foramen magnum with communicating vasculature visualized. CT: computed tomography; AVM: arteriovenous malformation.

A CT angiography (CTA) study of the head and neck demonstrated an arteriovenous malformation (AVM) rupture at the foramen magnum and cervicomedullary junction (CMJ) (Figure 2).

Neurosurgical consult revealed a poor prognosis as surgical candidacy was not appropriate for the case, and the patient was unfortunately pronounced brain dead.

## Discussion

Liver transplantation (LT) is the standard therapy for patients with acute and chronic liver failure of all etiologies. In the past year, over 8,000 LTs were performed, and LTs were the second most commonly performed organ transplantation [5]. Despite improvements to outcomes and long-term survival, a higher risk of cerebrovascular diseases in post-LT patients is a major point of concern [1]. Immunosuppression regimens and a prevalence of cardiovascular disease risk factors in the post-LT patient population may be significant contributors to the increased risk of cerebrovascular disease in LT recipients.

Following an LT, glucocorticoids are provided to patients for their immunosuppressive effects [6]. Immunosuppression in transplantation aims to dampen the organ recipient’s immune system to avoid transplant rejection by host immune cells while allowing for the newly transplanted organ to begin thriving in its new environment. This proves to be essential in both the short-term and long-term survivability of the transplanted organ, regardless of how well of an immunological match the organ donor and recipient are [7]. However, immunosuppressive agents are known to have major adverse side effects and predispose users to a variety of comorbidities, such as hypertension, diabetes, and neurotoxicity [4,8]. In particular, calcineurin inhibitors (CNIs), a class of commonly used immunosuppressant drugs, are known to be related to hypertension, nephrotoxicity, and an increased risk of developing chronic renal failure [4]. In a retroactive study examining the long-term medical complications of 139 long-term survivors of LTs, Sheiner et al. discovered diabetes and hypertension were more commonly found in LT recipients following a cyclosporine immunosuppressant regimen than in the general US population [8]. Cyclosporine, a common CNI-type immunosuppressant drug for LT recipients, may cause altered vascular reactivity, vasoconstriction, and impaired glomerular filtration, all factors possibly contributing to the increased prevalence of hypertension in post-LT patients [8].

Once oral medications can be administered post-transplant, patients are switched to taking prednisone 20 mg/day, tapered to 0 mg over three to six months. Unfortunately, steroids may contribute to a multitude of complications such as diabetes, fluid retention, and poor wound healing [9]. Thus, early steroid withdrawal after transplant has been described as advantageous to reduce side effects [10]. One meta-analysis published containing 16 randomized controlled trials compared corticosteroids alone to corticosteroids containing an immunosuppressive prescription post-transplant. Rejection events and kidney damage were more common in the group receiving corticosteroids alone, supporting the use of corticosteroids with immunosuppressive properties post-transplant [4]. Thus, while steroid administration may contribute to certain complications, they are still commonly administered to improve outcomes associated with the LT procedure.

Our patient experienced an AVM rupture three months after an unremarkable LT. Hereditary hemorrhagic telangiectasia (HHT) is the leading cause of AVMs. It is an autosomal dominant disorder with an estimated prevalence of one per 5,000 to 8,000 individuals [11]. The disorder, characterized by vascular dysplasia in various organs, may clinically manifest as epistaxis and gastrointestinal bleeding in addition to pulmonary and brain AVMs [12]. AVMs of the foramen magnum may also develop as a result of dural vein or sinus thrombosis and trauma; Chaudhary et al. speculated that the former variety of acquired AVM may result from an expansion of the dural arteries located in the sinus walls during thrombus development, subsequently creating an abnormal shunt [13]. Intracranial hemorrhages are significant causes of morbidity and death in patients with brain AVMs [13]. AVMs are thought to be comprised of tangled, dilated vascular structures that may contain abnormally high levels of angiogenic and fibrogenic factors as well as inflammatory cytokines [14]. These angiogenic factors, which include minor trauma, ischemia, delivery of exogenous growth factors, elevated endogenous angiogenic factors, inflammation, and infection, may also contribute to the development of AVMs [14].

AVM diagnosis is typically made with a combination of vascular imaging and either computed tomography (CT) or magnetic resonance imaging (MRI). MRI, in particular, can provide the location and topography of the AVM, moderating the next steps of a treatment plan [15]. In our case, we performed a CT angiography study, a modality notable for high sensitivity and specificity, to identify the ruptured AVM. Arteriovenous fistula (AVF) of the foramen magnum represented another differential diagnosis. Intracranial hemorrhage occurs in about 40 to 60% of brain AVMs, roughly 10% of which are located in the posterior fossa region [16]. In one study, 4.5% of treated AVMs were found to be skull base AVMs, representing nearly 40% of the dural AVMs [17]. Dural AVFs in the foramen magnum are quite rare, seen in about 1.5 to 4.2% of cranial shunting lesions, and may also present with posterior fossa intracranial hemorrhage [18,19]. The foramen magnum is very close to the spinal cord where the vascular anatomy can cause AVMs that resemble the one demonstrated in Figure 2.

AVM treatment may include surgical removal, endovascular treatment, stereotactic radiosurgery, and/or medical management for symptom relief. Notably, an AVM can still develop in the future after treatment. Interventional treatment aims to reduce the risks of AVM-related hemorrhaging and neurological complications. Patient age, comorbidities, AVM size and location, and venous drainage pattern are considered when deciding between medical management and interventional treatment [16]. Most patients with ruptured AVMs receive interventional treatment; however, interventions come with associated risks, including permanent neurological complications or death at a rate of 5 to 7.5% [16].

Unsurprisingly, patients with ruptured AVMs are found to be twice as likely to experience hemorrhage compared to patients with unruptured AVMs. AVMs are responsible for nearly 10% of SH; subarachnoid bleeding is particularly common in superficial AVMs [16]. Over roughly 50% of AVMs present with intracranial hemorrhage [15]. ICH accounts for 10 to 20% of all cerebrovascular events in the US, with nearly 50% of patients passing away in a medical facility [20]. Brain AVM rupture generally results in ICH and subarachnoid hemorrhage may occur alongside intraventricular hemorrhage, as demonstrated in our case. Acute ICH treatment includes the management of blood pressure, intracranial pressure, and managing abnormal coagulation. Risk factors for ICH include advanced age, hypertension, and amyloid beta-peptide sediments in blood vessels of the brain, known as cerebral amyloid angiopathy. ICH frequently presents with sudden neurological decline, diminished consciousness, seizures, and/or high blood pressure [20]. Neuroimaging, such as noncontract-CT or CT angiography must be done to differentiate ICH from other possible pathologies such as ischemic stroke, which have similar clinical presentations.

This case illustrates the crucial importance of recognizing aggravating factors of AVMs as well as rupture-related complications. With an uncomplicated LT three months prior in our patient, it is difficult to pinpoint the exact cause of the AVM development or rupture. Unfortunately, given our patient’s AVM rupture presenting as intraventricular hemorrhage, the prognosis was grim, and our patient was ultimately pronounced brain dead.

## Conclusions

While liver transplants (LTs) are life-saving procedures, serious health complications can be associated with the post-transplantation period. Usage of steroids, other immunosuppressants, and changes to hemodynamics may leave patients with an adverse risk of developing cerebrovascular complications. Notably, AVMs are a leading cause of death following a liver transplant. While they may be surgically addressed, intervention presents a new set of risks and considerations. In this report, we discuss a case of a post-LT patient with a ruptured cerebral AVM and associated extensive cerebral hemorrhaging. Consequently, the neurosurgical evaluation revealed an absence of brainstem reflexes and ultimately, the patient was pronounced brain dead.
